# Erosive Potential and Sugar Content of Popular Beverages: A Double Whammy for Dentition

**DOI:** 10.1155/2023/9924186

**Published:** 2023-02-15

**Authors:** Nik Mohd Mazuan Nik Mohd Rosdy, Noor Aina Shuhada Mohd Amin, Nurnabila Roslan

**Affiliations:** ^1^Centre of OMF Diagnostics and Medicine Studies, Faculty of Dentistry, Universiti Teknologi MARA, Sungai Buloh Campus, Shah Alam, Selangor, Malaysia; ^2^Faculty of Dentistry, Universiti Teknologi MARA, Sungai Buloh Campus, Shah Alam, Selangor, Malaysia

## Abstract

**Objectives:**

This study aims to identify the pH level and subsequently the erosive potential of beverages including their sugar content.

**Materials and Methods:**

Beverages were purchased from a local convenience store, and some of the beverages were freshly prepared. The acidity of each beverage was identified using a calibrated pH meter. The pH was obtained in triplicate, and the results were expressed in average values with standard deviations. The pH values were then used to determine their erosive potential and the sugar content was obtained from the packaging and recorded.

**Results:**

A total of 167 beverages were purchased and categorized. The beverages were categorized into 15 groups: milk tea, hawker drinks, instant drinks, fresh fruit juices, milk, energy drinks, designer coffee, soda, canned drinks, cultured milk, vegetable juices, cordials, bottled fruit drinks, tea, and mineral water. The pH value ranges from 2.65 to 7.85. Seven beverages (4.2%) were classified as extremely erosive, 53 beverages (31.1%) were classified as erosive, and 36 beverages (21.6%) were classified as minimally erosive. In total, 57.5% of the beverages were potentially erosive, and most of the soda and energy drinks were erosive. The highest sugar content per 100 g was BOH Teh Tarik Original (71.8 g), whereas the highest sugar content per serving was Carabao energy drink (10.8 g).

**Conclusion:**

High sugar content and low acidic content of beverages could have a negative impact on the dentition. An intervention is required from a public health perspective to regulate the consumption of sweetened and flavored beverages.

## 1. Introduction

### 1.1. Beverages

A beverage is a liquid for human consumption by drinking and beverages are categorized in many ways. Based on the Beverage Guidance Panel proposed by Varzakas et al. [1], beverages are ranked from the lowest to the highest value based on caloric and nutrient contents as well as related health benefits and risks. Drinking water was ranked as the preferred beverage to fulfill daily water needs, followed by tea and coffee, low-fat (1.5% or 1%) and skim (nonfat) milk and soy beverages, noncalorically sweetened beverages, beverages with some nutritional benefits (fruit and vegetable juices, whole milk, alcohol, and sports drinks), and calorically sweetened, nutrient-poor beverages [1]. On the other hand, sugar-sweetened beverages (SSB) are the mainstay when categorizing beverages. SSB is defined as any drink with caloric sweeteners, including carbonated soft drinks, sports drinks, energy drinks, fruit drinks, chocolate (or otherwise sweetened) milk, and sweetened coffee and tea, but not including 100 percent fruit juice or “diet” drink alternatives with noncaloric sweeteners [2].

### 1.2. Acidity in Beverages

Citric, phosphoric, ascorbic, malic, tartaric, oxalic, and carbonic acids are examples of dietary acids. Fruits and fruit juices, soft drinks, and vinegar, for example, contain these acids.

In addition, human observational studies have also found a link between tooth erosion and the consumption of a variety of acidic foods and beverages, such as fruit juices, soft drinks, vinegar, citrus fruits, and berries [3].

In general, a pH value of seven is neutral, while a pH of less than seven is acidic, and more than seven is alkaline. Citric acid, phosphoric acid, and other dietary acids are found in significant concentrations in some beverages, contributing to a lower pH value [1]. Thus, it has been recommended that malic acid be used instead of citric or phosphoric acid in some drinks because it causes less enamel damage than citric and phosphoric acids. Increasing the pH of drinks (>3.8) and reducing the titratable acidity may also significantly reduce the erosive potential [2]. According to recent research, low pH is the major predictor of beverage erosive potential, while calcium ion citrate chelation may contribute to erosion at higher pH [4].

### 1.3. Erosive Potential

Dental erosion occurs when the tooth enamel is directly exposed to acids, whereas caries is a condition caused by bacterial fermentation of carbohydrates in the oral cavity. Besides, it is also worth noting that, while erosion is largely a surface phenomenon, caries usually start as demineralization of enamel structure that eventually leads to a pit in the tooth surface [5]. Extrinsic acids, particularly acidic beverages, are a major cause of dental erosion (DE). Of all the parameters examined, the pH of a beverage appears to have the strongest effect on the beverage's propensity to promote tooth erosion. Various chemical characteristics of a potentially erosive substance have been found in the literature as possibly relevant in defining the erosive potential, including pH value, titratable acidity, buffering capacity, and Ca, Pi, and F concentrations [6].

### 1.4. Sugar Content in Beverages

Evidently, a report by Gwinn et al. emphasized that the daily sugar intake among Malaysian adolescents was still exceeding the range recommended by the WHO. In general, mono- and disaccharides are traditionally referred to as “sugars,” while purified sucrose is referred to as “sugar” or “refined sugar.” Additionally, the phrase “added sugars” is used by the American Heart Association (AHA) to describe sugars and syrups added to foods during processing or preparation, as well as sugars and syrups added at the table [4].

Sweetened or flavored beverages have become a daily delight for many of us. The reality behind this is that these beverages may negatively affect us, especially our dentition. While the public may be aware of the fact that excessive sweetened beverages consumption can cause dental caries and diabetes in general health and that the bacteria may consume sugar and further generate acid as by-products, many are not aware of the acidic nature of some beverages that could likewise harm their teeth. Direct action of the acidic environment will lead to dental erosion, which refers to the permanent loss of dental hard tissue (enamel and dentine) caused by acids etching away the enamel and dentine in a nonbacterial process.

Monosaccharides and disaccharides that are added to foods and drinks, as well as sugars found naturally in honey, syrups, fruit juices, and fruit juice concentrates, are all examples of free sugars [3]. Meanwhile, a sweetener refers to any naturally occurring or synthetically produced ingredient that gives food and beverages a sweet flavor. For instance, sucrose (table sugar) is the most popular sweetener in the food business and is considered the “gold” standard for sweet taste [1]. Sweet taste, in particular, is a source of pleasure as well as a sensory indication for energy [2]. Sugar also helps to balance the sweetness and acidity of fruit-based goods, including drinks, sauces, and preserves [4].

Currently, no data pertaining to the acid level of beverages are available in Malaysia, including awareness of the sugar content of the consumed beverages. These two important substances in the beverages may cause two different outcomes, which are dental erosion and dental caries. The beverages, which contain these two substances, may produce a significant effect on the dental tissue. Therefore, this study aims to identify the pH level and subsequently the erosive potential of beverages including their sugar content.

## 2. Materials and Methods

### 2.1. Beverage Categories, Acidity Profiling, and Relative Erosivity

We purchased commonly consumed beverages according to the listed types in Table 1. However, some of the beverages require preparation such as fruit and vegetable juices and cordials. We categorized these beverages into the following 15 categories: milk tea, hawker drinks, instant drinks, fresh fruit juices, milk, energy drinks, designer coffee, soda, canned drinks, cultured milk, vegetable juices, cordials, bottled fruit drinks, tea, and mineral water.

Fruit and vegetable juices were prepared fresh using 50 g of each fruit and vegetable and blended with 50 ml of distilled water. Meanwhile, for sweetened cordials that require dilution, approximately 50 ml of cordial was mixed with 100 ml of distilled water.

The pH value of the beverages was determined using the SevenEasy S20 pH meter (Mettler-Toledo Inc., Columbus, OH, USA). Approximately 50 ml of every beverage was sampled at a stabilized temperature of 22°C. The pH meter was calibrated using standardized buffer solutions with pH 2.0 and pH 10 prior to the first measurement. The reading was then obtained in triplicate, and the average values were calculated. Subsequently, we recorded the pH data as range and mean (standard deviation (SD)).

**Figure 1:**
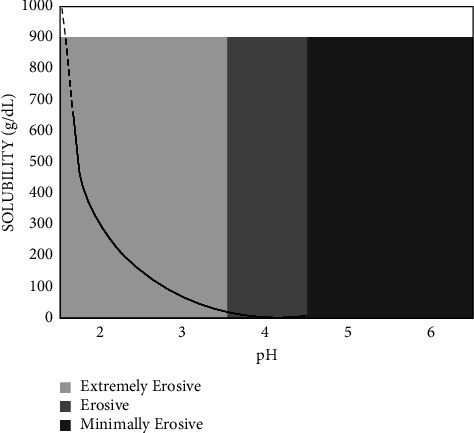
Erosion zones based on the theoretical solubility of apatite as a function of pH. g, grams; L, liters. Adapted with permission of Elsevier from Reddy et al. [7].)

We then determined the erosive potential of each beverage based on relative beverage erosivity zones from studies of apatite solubility in acid (Figure 1) by Chong et al. [7].

### 2.2. Sugar Content

We have also obtained information regarding the sugar content from the nutrition facts label on the packaged beverages. The sugar labeled as “Total Sugars (naturally occurring sugars)” and “Added Sugar (added to beverage/food during preparation)” on the packaging was considered [5]. However, beverages that were freshly prepared or purchased from hawkers did not contain sugar information; thus, there were no data available.

## 3. Results

### 3.1. Acidity Profiling and Characterizing of Relative Erosivity

We purchased and/or prepared 167 beverages and categorized them into 15 categories. The pH of every beverage was determined with a mean value and standard deviation (Table 1).

In terms of the acidity level, the Coca-Cola® Schweppes Bitter Lemon (pH = 2.65, SD = 0.01) was determined as the most acidic, while F&N Ice Mountain Natural Mineral Water (pH = 7.85, SD = 0.02) was the most alkaline in the Malaysian market. Each individual beverage pH and its erosive potential category are summarized in Table 1.

On the contrary, F&N Ice Mountain Natural Mineral Water has the highest pH (7.85, SD = 0.02). Beverages with pH above 6 include designer coffee, particularly their latte product, milk tea drink, milk, and some from instant drinks, milk, and vegetable juices. The bottled mineral water samples have pH values above 7 except for one brand, Sirma (pH: 6.5, SD = 0.02).

Seven beverages (4.2%) were categorized as extremely erosive, and most of the beverages are from the soda category, while 53 (31%) beverages were classified as erosive. Furthermore, all 14 beverages from the energy drink category are categorized as erosive, whereas minimally erosive beverages constituted only 36 (21.6%) beverages and showed pH values of 4–6. In addition, many of the beverages from cultured drinks, cordials, and bottled fruit drinks are likewise categorized as erosive, and these mostly include cultured milk and tea. Nonetheless, the remaining beverages are categorized as nonerosive (42.5%).

As summarized in Table 2, the lowest mean pH constituted soda (3.03, SD = 0.34), while there are 5 categories of beverages with a mean pH of <4 (categorized as erosive).

### 3.2. Sugar Content

We determined the sugar content from beverages per the package, and the sugar content was standardized into per 100 g/100 ml for comparison. Evidently, Boh Teh Tarik Original from the instant drink category has the highest sugar content per 100 g with 71.8 g, while Calpis Zero Orange and Calpis Zero Lychee flavors from the cultured drink category contained the lowest sugar content, which is only 1.5 g per 100 ml. These data are presented in Table 1. Based on the packaging of Boh Teh Tarik Original (1 serving—14.4 g), it contains almost 4 teaspoons of sugar. However, we were unable to compare all beverages due to the unavailability of data.

Based on WHO guidelines on sugar intake, 12 teaspoons (50 grams) of sugar per day for an adult is the maximum amount to consume. Our study showed that when consuming most of these drinks, at least 50% of the sugar intake comes from a single serving of the beverage.

## 4. Discussion

Issues related to beverages are discussed globally within and outside the public health concerns. However, many are unaware of the fact that the acidic content may also cause health hazards, especially to the dentition. At the same time, sugar is still widely discussed for its health impact. From a wider perspective, behavioral changes can be one of the indicators for the risk and health impacts; hence, these issues are discussed based on our findings.

### 4.1. Acidic Content and Erosive Potential

This study determines the pH of 167 beverages available in the Malaysian market and widely available across the nation. Specifically, we identified different types of beverages to cover almost all available beverages in the market and hawkers, since a wide beverage diversity in the market accounts for the large number of beverages consumed by the communities.

Our results are consistent with the beverage pH values reported by other investigators. For example, we indicated that the pH of Coca-Cola was 2.74 (SD = 0.02) (Table 1) compared to 2.61 [6], 2.37 [8], 2.39 [9], and 2.45 [10], while the pH of Schweppes tonic water was 2.73 (SD = 0.01) (Table 1) compared to 2.54 [8], 2.63 [6], and 2.48 [11]. Our findings are also similar to Schmidt and Huang's with regard to the lowest pH of 2.56 (SD = 0.01) for Pepsi®. This entails the same category as the most acidic beverages in our study (soda). [6].

One of the solutions to reduce the risk of dental erosion due to acidic beverages is calcium fortification [8]. Although the nutritional content of fruit drinks (bottled) could be valuable for health, the acidic content could supersede the health effect. In addition, the acidic effect could be buffered with the calcium fortification method in the beverages [6, 12]. The mechanism of the calcium in the beverages has been explained by Featherstone and Lussi [12]. Furthermore, most of our findings are also consistent with other studies with regard to the erosive potential of the beverages towards the tooth structure [13].

Fortified cultured milk is another example of a beverage with a low pH (4.0); however, its erosive effect is compensated with its high calcium and phosphate content as buffering properties [14]. Thus, frequent consumption of fortified cultured milk is presumably safer compared to the consumption of any other acidic beverages.

Teenagers are at higher risk of dental erosion because of the consumption of large amounts of acidic beverages [15]. Overall, our study found that more than 50% of the beverages were considered erosive (pH < 5.5). Thus, these beverages have the potential to cause dental problems among teenagers, which could generate oral impacts in later years.

Nonetheless, one of our study limitations is that sugar content could not be identified in many beverages that were not packaged beforehand. Besides, the laboratory method was also time-consuming. Therefore, further consideration should be taken to establish predictable and inexpensive models for determining the erosive potential of beverages.

### 4.2. Sugar-Sweetened Beverages

Since sugar is a global issue in public health, the World Health Organization (WHO) strongly recommends that the total sugar intake be 10% less than the total energy intake or less than 25 mg of free sugars per day [3]. In fact, some consumers are not aware of the sugar content because they do not read the nutritional information, and they may also be unaware of the type of sugar used in the ingredient. In general, beverages are labeled to have a high content of sugar when they have more than 22.5 g of total sugar per 100 g [16].

Based on the findings of our study, most of the beverages contained more than half sugar compared to the WHO's recommended intake. The number of instant drink consumption, for instance, is considerably high, and this is a common trend among students [17]. Instant drinks, especially premixed coffee powder, have the highest sugar level of all powdered drinks. Sucrose and lactose are the two most common sugars found in premixed beverages, with granulated sugar and creamer being the primary components [7].

### 4.3. Current Trend in Beverage Consumption

A study by Reddy et al. [18] in 2011 found that the frequency of energy drink consumption was higher among students of arts and sports as well as those who did not have breakfast on a regular basis, smoked cigarettes, drank alcoholic beverages, and who were regularly engaged in sports compared to medical students [18].

On the other hand, the consumption of 3-in-1 instant packets or drinks is surprisingly alarming, especially since they take less time to prepare and are widely available [19]. A study based on the Malaysian Adults Nutrition Survey (MANS) reported that the food consumption patterns of adults aged 18–59 years include tea (47%), coffee (28%), chocolate-based drinks (23%), and cordial syrup (11%) on daily basis [20]. Another study also found that 76% of Taiwanese college students between 19 and 22 years old consumed instant-packed coffee drinks at least once a week [21]. Furthermore, ready-to-drink packaged coffee beverages were also more popular than freshly brewed or instant coffee. Their criteria for selecting this option include variations in flavor, brands or promotion, and price or volume variables [21].

Nevertheless, since our study has implications for further research, it is necessary to develop strategies in which positive attitudes can be converted into promising practices. Besides serving as a reference for future investigations, this study should also act as an immediate resource for health practitioners and individuals to facilitate dietary counseling and guide consumers or patients as healthy dietary decisions.

## 5. Conclusion

Overall, the study found that a high percentage of beverages in Malaysia have the potential to cause dental erosion. Most beverages, including some bottled water, have been found to be acidic. Thus, the risk of dental erosion by the consumption of the tested beverages poses oral and general health risks for the public, which warrants further investigation.

In essence, beverages that contain both acidic and high sugar content shall be well-informed and acknowledged by the relevant authorities so that appropriate measures to intervene in the issues can be taken. The interventions may include strengthening the awareness program in beverage consumption for dental and general health.

## Figures and Tables

**Table 1:** Sugar content (packaged), pH, and relative erosivity of the beverages by category.

Brand	Model	Sugar content (per 100 g)	pH		Erosive potential
			Mean	SD	
*Milk tea drinks*					
Chatime	Milk Tea	NA	6.40	0.01	Not erosive
Coolblog	Brown Sugar Milk Tea	NA	6.55	0.03	Not erosive
Tiger sugar	Brown Sugar Pearl Milk with Cream	NA	6.58	0.02	Not erosive
Daboba	Brown Sugar Milk Tea	NA	6.63	0.03	Not erosive
Family Mart	Brown Sugar Bubble Milk	NA	6.67	0.01	Not erosive
Gong Cha	Oolong Tea	NA	6.67	0.02	Not erosive
Koi	Brown Sugar Milk Tea	NA	6.77	0.02	Not erosive
Bubblebee	Honey Milk with Black Pearl	NA	6.84	0.01	Not erosive
Secret recipe	Boba brown Sugar Milk Tea	NA	6.89	0.01	Not erosive
Tealive	Bang Bang Milk Tea	NA	7.01	0.01	Not erosive

*Hawker drinks*					
Asam Boi	NA	NA	3.02	0.00	Erosive
Orange	NA	NA	3.17	0.01	Erosive
Sea coconut	NA	NA	3.75	0.02	Erosive
Guava and Asam Boi	NA	NA	4.12	0.01	Minimally erosive
Watermelon	NA	NA	4.89	0.02	Minimally erosive
Coconut	NA	NA	5.73	0.01	Not erosive
Thai tea	NA	NA	6.36	0.01	Not erosive
Honeydew	NA	NA	6.55	0.01	Not erosive
Green tea	NA	NA	6.98	0.01	Not erosive
Corn	NA	NA	7.12	0.01	Not erosive

*Instant drinks*					
Anramin	Lemon Tea	NA	2.88	0.03	Extremely erosive
Nescafe	Blend & Brew	60.0	6.32	0.01	Not erosive
Wilson class	Premix Tongkat Ali Ginseng Coffee	NA	6.35	0.02	Not erosive
Nescafe	Dark Latte	45.0	6.49	0.02	Not erosive
Radix	Premix Coffee	NA	6.54	0.01	Not erosive
Boh	Teh Tarik Original	71.8	6.55	0.02	Not erosive
Alicafe Warung	Klasik 3 Dalam 1	NA	6.58	0.01	Not erosive
Adabi	Kopi 5 in 1-Tongkat Ali and Cordyceps	NA	6.70	0.01	Not erosive
Oligo	Coklat belgium	65.3	6.95	0.02	Not erosive
Alitea Warung	Teh Tarik 3 Dalam 1	NA	7.20	0.02	Not erosive
*Fresh fruit juices*					
Kiwi	NA	NA	3.58	0.01	Erosive
Strawberry	NA	NA	3.65	0.01	Erosive
Apple	NA	NA	3.66	0.01	Erosive
Orange	NA	NA	3.86	0.01	Erosive
Guava	NA	NA	4.06	0.01	Minimally erosive
Tomato	NA	NA	4.64	0.02	Minimally erosive
Dragon fruit	NA	NA	5.05	0.01	Minimally erosive
Papaya	NA	NA	5.50	0.01	Minimally erosive
Watermelon	NA	NA	6.01	0.01	Not erosive
Pumpkin	NA	NA	6.56	0.02	Not erosive
Avocado	NA	NA	6.62	0.02	Not erosive

*Milk*					
Nestle	Bear brand milk	4.8	6.47	0.02	Not erosive
Dutch Lady	Purefarm low-fat high-calcium milk	0.0	6.53	0.01	Not erosive
Dutch Lady	Kurma	6.7	6.57	0.01	Not erosive
Dutch Lady	100% fresh milk	2.6	6.61	0.01	Not erosive
Dutch Lady	Full cream milk	4.0	6.67	0.01	Not erosive
Goodday	Banana flavored milk	6.7	6.69	0.02	Not erosive
Dutch Lady	Coffee	5.0	6.72	0.01	Not erosive
Dutch Lady	Strawberry flavored milk	6.6	6.76	0.03	Not erosive
Lactasoy	Soy milk chocolate	9.6	6.86	0.03	Not erosive
Pokka	Melon milk	9.9	6.95	0.01	Not erosive
Pokka	Mango milk	10.5	7.07	0.01	Not erosive

*Energy drinks*					
Livita	Honey	4.8	3.14	0.01	Erosive
KF	Kacip Fatimah Herbal Beverage	10.1	3.34	0.01	Erosive
OK	Longjack	12.4	3.34	0.01	Erosive
Orang Kuat	Tongkat Ali Extra Honey	5.0	3.43	0.01	Erosive
Redbull	25% less sugar	12.3	3.53	0.01	Erosive
Black	Black Energy Drink Mike Tyson Original Premium	10.0	3.55	0.01	Erosive
Power Root	Tongkat Ali and Honey Dates	11.2	3.56	0.01	Erosive
Revive	Original Isotonic Drink	4.9	3.57	0.02	Erosive
Redbull	360 Energy Pro	16.8	3.60	0.01	Erosive
All WiiNS	Strong energy drink	4.6	3.61	0.01	Erosive
Redbull	Sugar free with taurine	0.0	3.64	0.01	Erosive
F&N	100plus isotonic drink	4.9	3.69	0.01	Erosive
Redbull	Plus Slim Can	0.0	3.74	0.00	Erosive
Carabao Tawandang Co. Ltd.,	Carabao energy drink	17.2	3.83	0.03	Erosive

*Designer coffee*					
Tealive	Latte	NA	6.28	0.01	Not erosive
Secret recipe	Latte	NA	6.34	0.01	Not erosive
Costa	Latte	NA	6.38	0.01	Not erosive
Union artisan coffee	Latte	NA	6.44	0.02	Not erosive
Dunkin donut	Latte	NA	6.48	0.01	Not erosive
Mcdonald	Latte	NA	6.52	0.00	Not erosive
Sans Francisco	Latte	NA	6.53	0.01	Not erosive
O'Briens	Latte	NA	6.57	0.01	Not erosive
Cbtl	Latte	NA	6.66	0.01	Not erosive
Starbucks	Latte	NA	6.68	0.01	Not erosive

*Soda*					
The Coca-Cola company	Schweppes Bitter Lemon	8.8	2.65	0.01	Extremely erosive
PepsiCo	Pepsi	4.9	2.70	0.01	Extremely erosive
The Coca-Cola company	Schweppes tonic water	8.9	2.73	0.01	Extremely erosive
The Coca-Cola company	Coca-Cola	4.6	2.74	0.02	Extremely erosive
F&N	Ice cream soda	4.9	2.81	0.01	Extremely erosive
PepsiCo	Mountain dew	4.8	3.03	0.01	Erosive
The Coca-Cola company	Sprite	4.6	3.30	0.01	Erosive
F&N	Sarsi	2.5	3.38	0.02	Erosive
A&W	Sarsaparilla	4.7	3.46	0.02	Erosive
Kickapoo	Joy juice	4.9	3.47	0.04	Erosive

*Canned drinks*					
Yeo's	Lychee	4.9	3.17	0.07	Erosive
F&N	Seasons Ice Lemon Tea	4.4	3.21	0.03	Erosive
F&N	Seasons Buah Kundur Less Sugar	5.3	3.78	0.04	Erosive
Pearl Kacip Fatimah	Kacip Fatimah & Kolagen	11.0	3.78	0.03	Erosive
Rida	Pineapple juice	9.8	4.09	0.01	Minimally erosive
Yeo's	Winter Melon Tea (less sugar)	4.9	4.35	0.04	Minimally erosive
Yeo's	Air Tebu	4.9	4.74	0.08	Minimally erosive
Yeo's	Coconut juice	9.0	5.18	0.07	Minimally erosive
Yeo's	Sarang Burung	4.9	5.86	0.17	Not erosive
F&N	Water Chestnut	4.9	5.93	0.03	Not erosive
Nestle	Nescafe Cold Brew Hazelnut	4.8	6.36	0.01	Not erosive
Nestle	Nescafe latte	4.6	6.48	0.03	Not erosive
OldTown White Coffee	OldTown White Coffee Classic	NA	6.52	0.02	Not erosive
Nestle	Nescafe Ipoh White Coffee	6.8	6.58	0.14	Not erosive
Yeo's	Cincau jelly	4.9	6.63	0.08	Not erosive
Nestle	Milo Actigen Mocha	6.8	6.64	0.01	Not erosive

*Cultured milk*					
Yakult	Ace	14.0	3.61	0.01	Erosive
Calpis	Zero orange flavor	1.5	3.74	0.01	Erosive
Yobick	Mulberry blueberry	11.1	3.74	0.01	Erosive
Calpis	Zero lychee flavor	1.5	3.74	0.01	Erosive
Calpis	Sour delight	6.9	3.80	0.00	Erosive
Yeo's	Yeogurt grape	4.9	3.88	0.02	Erosive
Vitagen	Apple	8.5	4.02	0.02	Minimally erosive
Vitagen	Grape	10.2	4.05	0.02	Minimally erosive
Vitagen	Orange	10.3	4.10	0.02	Minimally erosive
Vitagen	Original	12.2	4.12	0.02	Minimally erosive
Lactel	Bliss tropica	8.1	4.25	0.01	Minimally erosive
Lactel	Bliss mango	8.1	4.26	0.01	Minimally erosive

*Vegetable juices*					
Aloe vera	NA	NA	4.27	0.01	Minimally erosive
Beetroot	NA	NA	5.82	0.02	Not erosive
Cucumber	NA	NA	5.82	0.23	Not erosive
Celery	NA	NA	6.00	0.02	Not erosive
Cabbage	NA	NA	6.02	0.02	Not erosive
Carrot	NA	NA	6.18	0.01	Not erosive
Kale	NA	NA	6.22	0.04	Not erosive
Spinach	NA	NA	6.44	0.02	Not erosive
Broccoli	NA	NA	6.75	0.01	Not erosive
Swiss Chard	NA	NA	7.24	0.01	Not erosive

*Cordials*					
PotonGuler Keto	Sugar free syrup	0.0	2.86	0.01	Extremely erosive
Sunquick	Tropical	7.5	3.14	0.01	Erosive
F&N	Anggur	11.7	3.14	0.03	Erosive
F&N	Orange	10.1	3.19	0.01	Erosive
Sunquick	Ribena	9.0	3.23	0.01	Erosive
Sunquick	Exotic	7.5	3.25	0.02	Erosive
Sunquick	Mangga	7.5	3.43	0.01	Erosive
F&N	Root beer	10.7	3.95	0.01	Erosive
F&N	Sarsi	10.7	4.03	0.04	Minimally erosive
F&N	Syrup	10.8	4.65	0.05	Minimally erosive

*Bottled fruit drinks*					
Green love	Sour plum	4.9	3.20	0.01	Erosive
Pran	Apple	4.5	3.28	0.02	Erosive
Latina	Pineapple	4.5	3.35	0.01	Erosive
Latina	Pomegranate	4.7	3.40	0.02	Erosive
Latina	Mango	4.7	3.48	0.01	Erosive
Tropicana twister	Fruit pulp orange	10.6	3.51	0.06	Erosive
Minute maid	Orange	4.1	3.64	0.00	Erosive
Minute maid	Tropical	4.2	3.66	0.01	Erosive
Best	Mango nectar	12.8	3.73	0.02	Erosive
Marigold	Soursop	8.0	3.96	0.02	Erosive
Marigold	Pink guava	9.9	4.12	0.02	Minimally erosive

*Tea*					
Ahmad tea London	Ceylon tea	NA	4.94	0.01	Minimally erosive
Dilmah	Elegant Earl Grey	NA	4.96	0.02	Minimally erosive
Cameron valley	Lemon flavoured tea	NA	5.02	0.01	Minimally erosive
Dilmah	Rose with French vanilla	NA	5.05	0.02	Minimally erosive
Boh	Cameronian Gold Blend	NA	5.08	0.01	Minimally erosive
Tehran's tea	Oolong tea	NA	5.21	0.03	Minimally erosive
Harney & Sons	Egyptian chamomile	NA	5.51	0.01	Not erosive
Harney & Sons	Jasmine fragrant green tea	NA	5.54	0.01	Not erosive
Dilmah	Ceylon green tea	NA	5.56	0.01	Not erosive
Lipton	Green tea	NA	5.73	0.01	Not erosive
Lipton	Peppermint	NA	6.08	0.01	Not erosive
Pickwick	Camomile	NA	6.35	0.01	Not erosive

*Mineral water*					
Sirma	Natural mineral water	NA	6.50	0.02	Not erosive
Desa	Natural mineral water	NA	7.01	0.01	Not erosive
KK mart	Natural mineral water	NA	7.01	0.02	Not erosive
Spritzer	Natural mineral water	NA	7.18	0.03	Not erosive
Evafresh	Natural mineral water	NA	7.31	0.01	Not erosive
Ombak	Natural mineral water	NA	7.35	0.02	Not erosive
Cactus	Natural mineral water	NA	7.45	0.02	Not erosive
Anda	Natural mineral water	NA	7.50	0.05	Not erosive
myNEWS	Natural mineral water	NA	7.82	0.02	Not erosive
F&N Ice Mountain	Natural mineral water	NA	7.85	0.02	Not erosive

**Table 2:** Beverage type descriptive statistics.

Beverage type	Number of beverage (*n* = 167)	pH range	Mean pH
Milk tea drinks	10	6.40–7.01	6.70 (0.18)
Hawker drinks	10	3.02–7.12	5.17 (1.58)
Instant drinks	10	2.88–7.20	6.25 (1.22)
Fresh fruit juices	11	3.65–6.62	4.83 (1.18)
Milk	11	6.47–7.07	6.72 (0.18)
Energy drinks	14	3.14–3.83	3.54 (0.18)
Designer coffee	10	6.28–6.68	6.49 (0.13)
Soda	10	2.65–3.47	3.03 (0.34)
Canned drinks	16	3.17–6.63	5.21 (1.32)
Cultured milk	12	3.61–4.26	3.94 (0.22)
Vegetable juices	10	4.27–6.75	6.08 (0.77)
Cordials	10	2.86–4.65	3.49 (0.55)
Bottled fruit drinks	11	3.20–4.12	3.58 (0.28)
Tea	12	4.94–6.35	5.42 (0.46)
Mineral water	10	6.50–7.85	7.30 (0.40)

## Data Availability

The data used to support the findings of this study are available from the corresponding author upon request.

## References

[1] Varzakas T., Labropoulos A., Anestis S. (2012). *Sweeteners: Nutritional Aspects, Applications, and Production Technology*.

[2] Spillane W. J. (2006). *Optimising Sweet Taste in Foods*.

[3] Organization W. H. (2015). *Guideline: Sugars Intake for Adults and Children*.

[4] Gwinn R. (2013). Industry position papers-technology and ingredients to assist with the reduction of sugar in food and drink.

[5] Azaïs-Braesco V., Sluik D., Maillot M., Kok F., Moreno L. A. (2017). A review of total & added sugar intakes and dietary sources in Europe. *Nutrition Journal*.

[6] Schmidt J., Huang B. (2020). The Acidity of Non-alcoholic Beverages in Australia: Risk of Dental Erosion. *International Journal of Scientific Study*.

[7] Chong C. P., Haron H., Shahar S., Md Noh M. F. (2019). Individual sugars contents in cooked dishes, processed foods, fruits and beverages commonly consumed by Malaysian. *Journal of Food Composition and Analysis*.

[8] Reddy A., Norris D. F., Momeni S. S., Waldo B., Ruby J. D. (2016). The pH of beverages in the United States. *Journal of the American Dental Association*.

[9] Cochrane N., Cai F., Yuan Y., Reynolds E. (2009). Erosive potential of beverages sold in Australian schools. *Australian Dental Journal*.

[10] Cochrane N., Yuan Y., Walker G. (2012). Erosive potential of sports beverages. *Australian Dental Journal*.

[11] Larsen M., Nyvad B. (1999). Enamel erosion by some soft drinks and orange juices relative to their pH, buffering effect and contents of calcium phosphate. *Caries Research*.

[12] Featherstone J., Lussi A. (2006). Understanding the chemistry of dental erosion. *Dental erosion*.

[13] Wegehaupt F., Günthart N., Sener B., Attin T. (2011). Prevention of erosive/abrasive enamel wear due to orange juice modified with dietary supplements. *Oral Diseases*.

[14] Lussi A., Megert B., Peter Shellis R., Wang X. (2012). Analysis of the erosive effect of different dietary substances and medications. *British Journal of Nutrition*.

[15] Rios D., Honório H. M., Magalhães A. C., Wiegand A., de Andrade Moreira Machado M. A., Buzalaf M. A. R. (2009). Light cola drink is less erosive than the regular one: an in situ/ex vivo study. *Journal of Dentistry*.

[16] Yusta-Boyo M. J., Bermejo L. M., García-Solano M. (2020). Sugar content in processed foods in Spain and a comparison of mandatory nutrition labelling and laboratory values. *Nutrients*.

[17] Gan W. Y., Mohamed S. F., Law L. S. (2019). Unhealthy lifestyle associated with higher intake of sugar-sweetened beverages among Malaysian school-aged adolescents. *International Journal of Environmental Research and Public Health*.

[18] Attila S., Çakir B. (2011). Energy-drink consumption in college students and associated factors. *Nutrition*.

[19] Changkari A., Ravindran P. (2013). *A study on food quality dimensions of 3 in 1 instant packet drink*.

[20] Norimah A. K., Safiah M., Jamal K. (2008). Food consumption patterns: findings from the Malaysian adult nutrition Survey (MANS). *Malaysian Journal of Nutrition*.

[21] Hsu J. L., Hung W. C. (2005). Packed coffee drink consumption and product attribute preferences of young adults in Taiwan. *Food Quality and Preference*.

